# Feline herpesvirus infection and pathology in captive snow leopard

**DOI:** 10.1038/s41598-022-08994-4

**Published:** 2022-04-28

**Authors:** Qiaoxing Wu, Hongchao Wu, Shunfu He, Yuxiu Liu, Yalei Chen, Xinzhang Qi, Xiangyang Gu, Yifan Wen, Xuelin Jin, Yipeng Jin, Kegong Tian

**Affiliations:** 1 Shaanxi Institute of Zoology, Xi’an, 710032 China; 2 College of Animal Science and Veterinary Medicine, Henan Agricultural University, Zhengzhou, 450046 China; 3 National Research Center for Veterinary Medicine, Luoyang, 471003 China; 4 Qinghai-Tibet Plateau Wild Zoo, Xining, 810008 China; 5 Huadong Medicine Co., Ltd, Hangzhou, 310011 China; 6 Chengdu Research Base of Giant Panda Breeding, Chengdu, 610057 China; 7 College of Veterinary Medicine, China Agricultural University, Beijing, 100193 China

**Keywords:** Pathogens, Herpes virus

## Abstract

Feline herpesvirus type 1 (FHV-1) is a common causative agent of domestic cats’ rhinotracheitis in domestic cats, and it increasingly threatens wild felids worldwide. The endangered snow leopard (*Panthera uncia*) belongs to the family Felidae, and it is the top predator on the Tibetan Plateau. Here we report the identification and isolation of FHV-1 from three dead captive snow leopards that presented with sneezing and rhinorrhea. To explore the relationship between FHV-1 and their deaths, organs and nasal swabs were collected for histopathology, viral isolation and sequence analysis. The results revealed that all three snow leopards were infected with FHV-1. The first animal died primarily of cerebral infarction and secondary non-suppurative meningoencephalitis that was probably caused by FHV-1. The second animal died mainly of renal failure accompanied by interstitial pneumonia caused by FHV-1. The cause of death for the third animal was likely related to the concurrent reactivation of a latent FHV-1 infection. The gD and gE gene sequence alignment of the isolated FHV-1 isolate strain revealed that the virus likely originated from a domestic cat. It was found that FHV-1 infection can cause different lesions in snow leopards than in domestic cats and is associated with high risk of disease in wild felids. This suggests that there should be increased focus on protecting wild felids against FHV-1 infections originating from domestic cats.

## Introduction

Infection with feline herpesvirus type 1 (order *Herpesvirals*, family *herpesviridae*, genus *Varicellovirus,* species *Felid herpesvirus 1;* FHV-1), which is known as the causative agent of feline viral rhinotracheitis, is widespread in domestic cats^1^. FHV-1 infection is often fatal to kittens; however, adult cats can generally survive and maintain a lifelong latent infection^2^. The initial clinical symptoms of FHV-1 infecion in feline hosts are conjunctivitis, keratitis, and upper respiratory disease, with pneumonia as an occasional complication^3^. FHV-1 is mainly transmitted through direct contact between infected and susceptible animals, and vertical transmission has not yet been reported^4^. Although domestic cats are the main hosts of FHV-1, several cases of FHV-1 infections in wildlife have been recently reported^5–7^. FHV-1 infections have been identified in European wildcats (*Felis silvestris silvestris*), sand cats (*Felis margarita*), leopard cats (*Felis bengalensis*), cheetahs (*Acinonyx jubatus*), mountain lions (*Puma concolor*), little spotted cats (*Leopardus tigrinus*), margays (*Leopardus wiedii*), ocelots (*Leopardus pardalis*), jaguarundis (*Herpailurus yaguarondi*), jaguars (*Panthera onca*), and south China tigers (*Panthera tigris amoyensis*) by serological or molecular methods^8–12^. In one example, a South China tiger that died of an FHV-1 infection presented with the clinical signs of excessive salivation, sneezing and purulent nasal discharge^6^.

The snow leopard (*Panthera uncia*, family *Felidae*) is an endangered felid and a top predator on the Tibetan Plateau and in the surrounding mountain ranges^13^. Its range includes Afghanistan, Bhutan, China, India, Kazakhstan, Kyrgyzstan, Mongolia, Nepal, Pakistan, Russia, Tajikistan, and Uzbekistan. It is estimated that there are only approximately 4000–7000 individual snow leopards currently living in the wild (Snow Leopard Network, 2014). Chinese snow leopards are primarily distributed across Qinghai Province and the Tibet and Xinjiang Autonomous Regions. They are also found in Gansu, Sichuan and Yunnan Provinces and the Inner Mongolia Autonomous Region^4^. Over the past two decades, significant progress has been achieved in the conservation of snow leopards in China and poaching has been effectively curbed. Despite this, infectious diseases, such as canine distemper^14^, carried by domestic pets have become a new threat to snow leopards, because: (1) the number of stray dogs and cats that have been lost or abandoned near wildlife refuges has increased, and (2) owners increasingly bring their pets into reserves when travelling. Because many owners do not regularly immunize their pets, the incidence rates of infection of common infectious diseases in dogs and other pets are high^15^.

In December 2019, three snow leopards that were housed within 40 m of each other in the Qinghai-Tibet Plateau Wild Zoo respectively exhibited symptoms of sneezing and rhinorrhea in the same week. Nasal swabs were collected and tested to detect potential pathogens such as canine distemper virus (CDV), FHV-1, feline infectious peritonitis virus (FIPV), feline panleukopenia virus (FPLV), feline leukaemia virus (FeLV), feline calicivirus (FCV), mycoplasma and chlamydia using real-time fluorescence-based quantification polymerase chain reaction (real-time qPCR). Brain, spleen and kidney tissue samples were detected for the severe fever with thrombocytopenia syndrome virus (SFTSV, which is a novel bunyavirus and tick-borne zoonotic pathogen) using PCR^16^. Three nasal swabs were positive for FHV-1. Furthermore, virus isolation, immunohistochemistry, and other methods were used to analyse the characteristics of the strain of FHV-1 identified in the snow leopards. This report primarily confirm that infection with FHV-1 resulted in the deaths of these captive snow leopards and describes the pathology.

## Results

In general, the cause of death in animals is determined by the following process: the examination of the clinical symptoms, the performance of a necropsy and a histopathological examination, and finally the identification of the suspected pathogens based on the results of the preceding investigations. In this study, histological examinations of formalin-fixed tissues were performed, with samples taken from the brain and tonsils of Case 1 and the lung, heart, liver, spleen, and kidney of Case 2.

### Clinical information and pathological diagnosis

#### Case 1

Case 1 was a male snow leopard with cataracts aged over 10 years. This was the first animal to exhibit symptoms of sneezing and rhinorrhea and it subsequently presented with convulsions before dying (Table 1). The course of the illness lasted for one week.

**Table 1:** The clinical information and pathogen examination results of three snow leopards.

Snow leopards	Case 1	Case 2	Case 3
Age	> 10y	> 10y	~ 10y
Gender	Male	Female	Male
Background disease	Cataract	The right leg fracture	ND
Clinical symptoms	Sneezing, rhinorrhea and convulsion	Sneezing, rhinorrhea and anuria	Sneezing and rhinorrhea
Outcome	Dead	Dead	Recovery but died 5 month later
**Detection of suspicious pathogens**			
CDV	−	−	−
FHV-1	+	+	+
FIPV	−	−	−
FPLV	−	−	−
FeLV	−	−	−
FCV	−	−	−
Mycoplasma	−	−	−
Chalmydia	−	−	−
SFTSV	−	−	−
**Detection of FHV-1**			
Nasal swab	+	+	+
Tonsil	−	ND	ND
Lung	+	+	ND
liver	−	−	ND
Spleen	−	−	ND
Kidney	−	−	ND
Brain	−	ND	ND

+, positive; −, negative; ND, No data. The method of detection of suspicious pathogens is real-time qPCR. The method of detection of FHV-1 in tissues is PCR.

**Figure 1:**
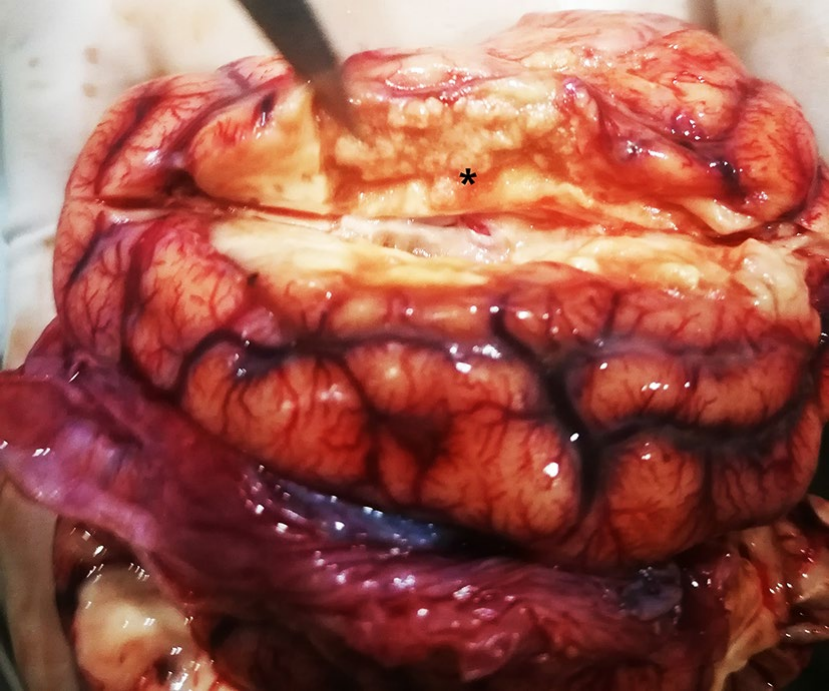
Gross observation of brain of Case 1. Meningeal congestion and a region of liquefactive necrosis in the right cerebral hemisphere with an area of approximately 2 cm × 0.5 cm × 1 cm are observed (*).

**Figure 2:**
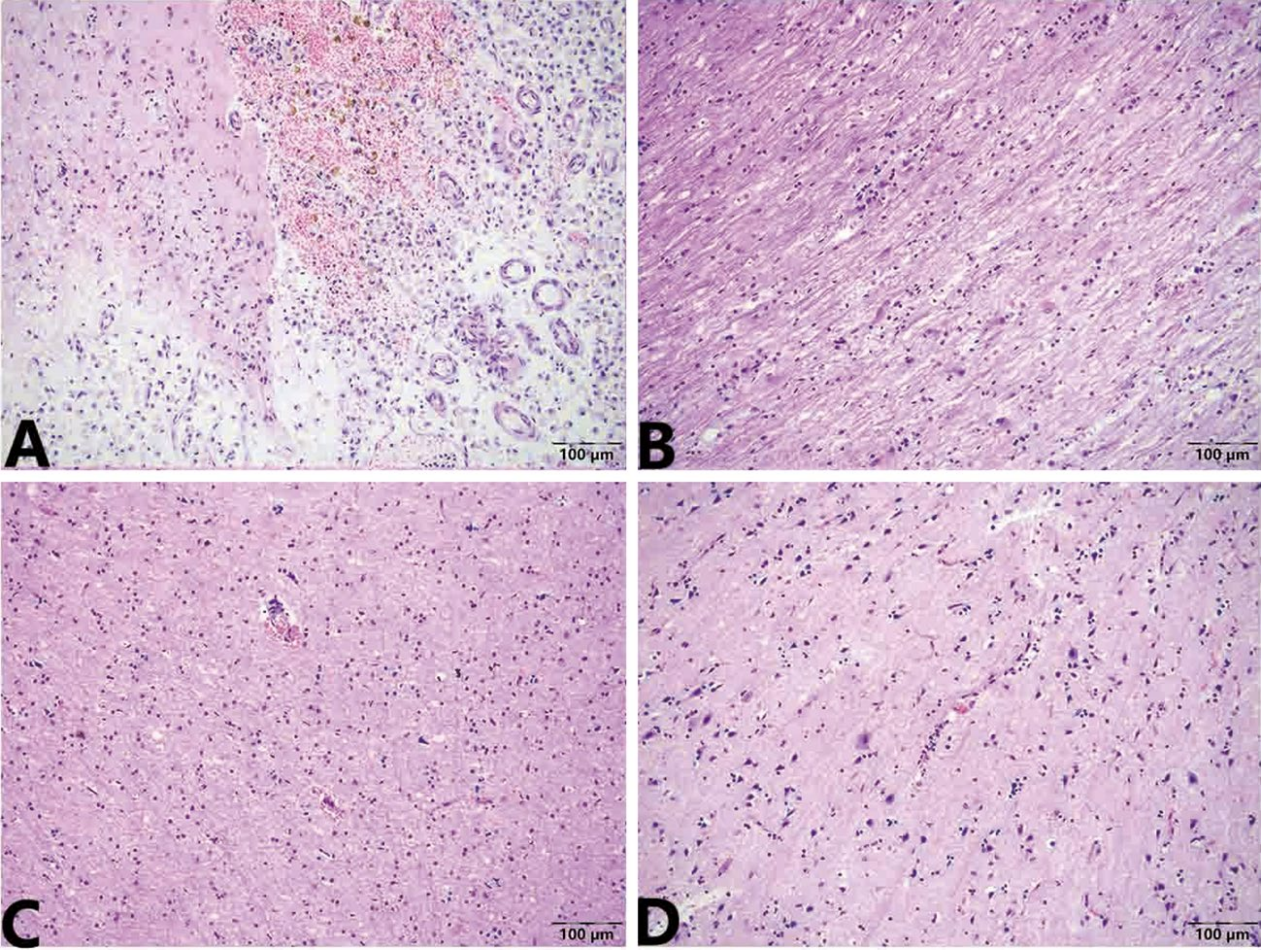
Microscopic change of brain of Case 1. (**A**) The lesions of hyperemia and haemorrhage, liquefactive necrosis, a massive collection of foam cells, and haemosiderosis are presented in the meninges and cerebral cortex. (**B**) Demyelination reactions are visible in white matter. (**C**) Bleeding foci scattered in cortex. (**D**) Capillary hyperemia; edema; moderate vascular cuff reaction (lymphohistiocytic perivascular infiltrates), neuron necrosis and neuronophagia are also visible in the brain parenchyma. Stained using haematoxylin and eosin (H&E). Captured by Olympus CX 43 microscope and EPview Ver 1.2. olympus-sis.com.

During the necropsy, meningeal congestion and a region of liquefactive necrosis in the right cerebral hemisphere with an area of approximately 2 cm × 0.5 cm × 1 cm were observed (Fig. 1). Moreover, no obvious abnormalities was observed in the other organs. The results of the histopathological examination indicated the presence of meningeal hyperaemia and haemorrhage, liquefactive necrosis in the cerebral cortex, a massive collection of foam cells, and heamosiderosis. Capillary hyperaemia, bleeding foci, oedema, vascular cuff reaction (lymphohistiocytic perivascular infiltrates), neuronal necrosis, neuronophagia, and demyelination reaction were visible in the brain parenchyma (Fig. 2).

The foam cells and haemosiderosis indicated that the cerebral infarction was old^17,18^. The meningoencephalitis was in the acute stage and an obvious demyelination reaction was visible in the white matter, which was supported by clinical neural symptom. Therefore, the pathological diagnosis was old cerebral infarction and secondary non-suppurative meningoencephalitis.

#### Case 2

Case 2 was a female snow leopard aged over 10 years that had a right leg fracture that had been treated with surgery. It was the second animal to present with sneezing and rhinorrhea 2 days after symptom onset in Case 1. This female snow leopard died one week after first showing symptoms and exhibited anuria for the final 2 days.

**Figure 3:**
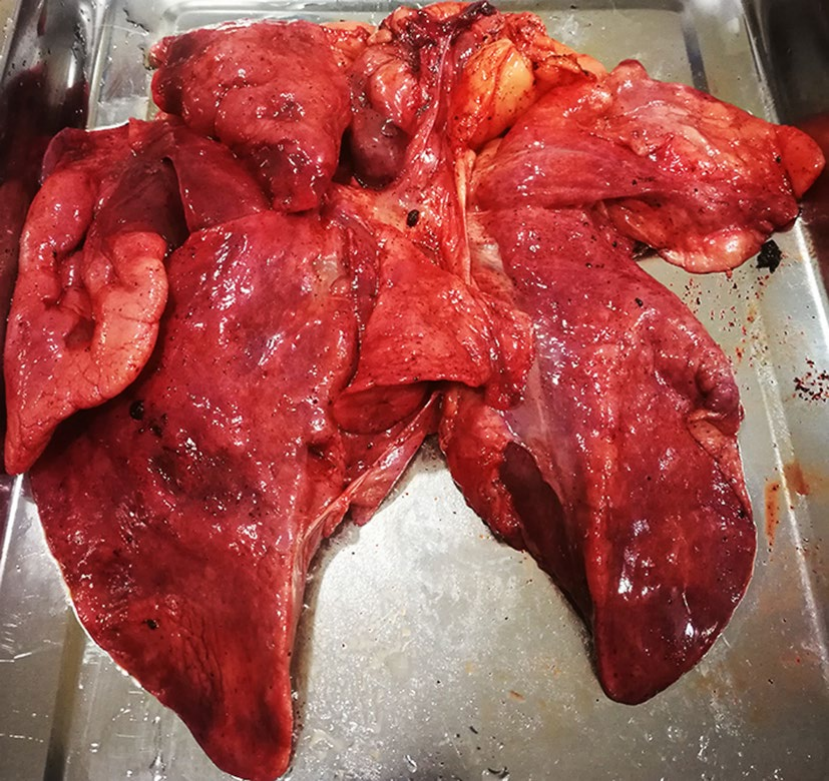
Gross observation of lung of Case 2. The lung presents dull-red, swelling, and liquid leakage from the section.

**Figure 4:**
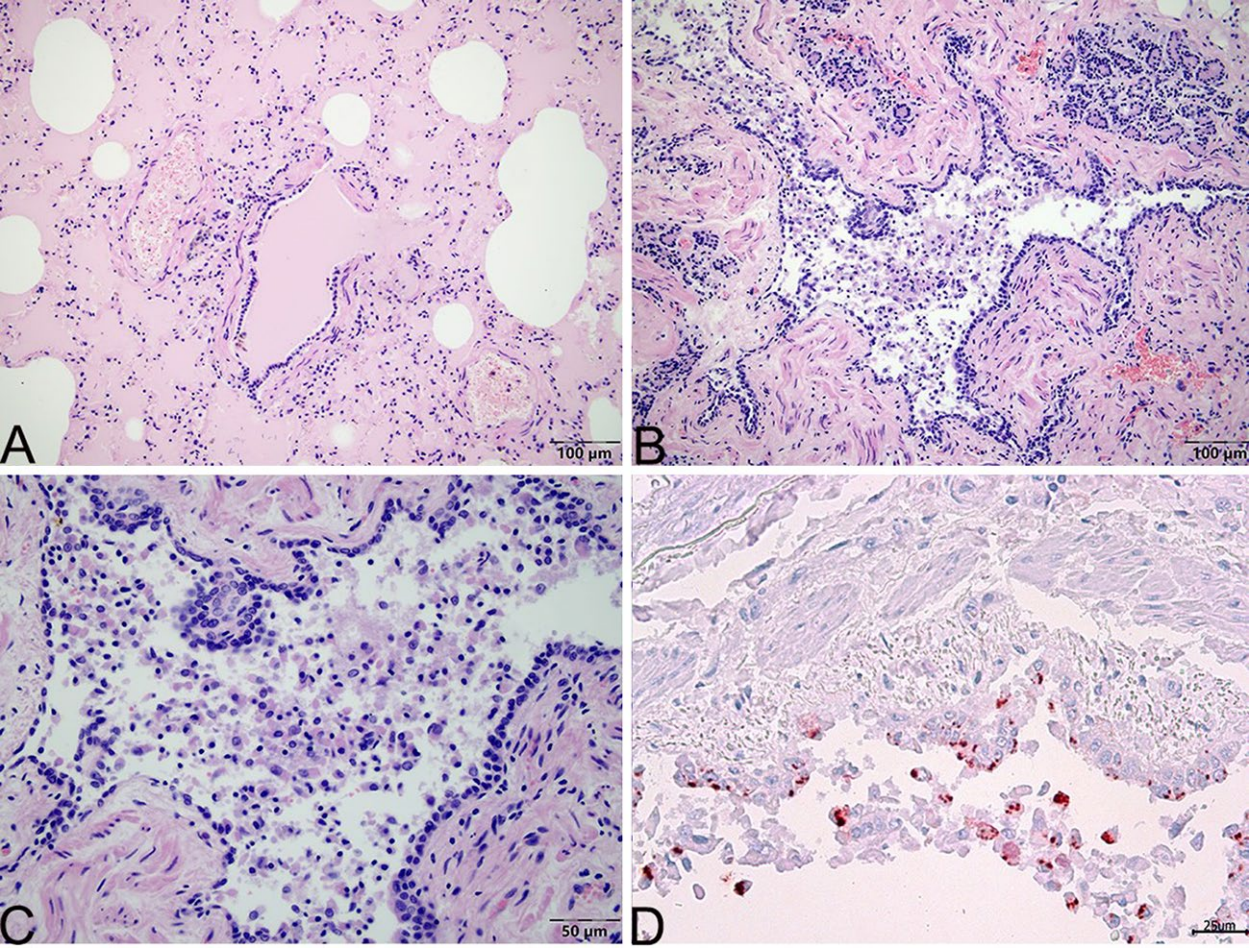
Microscopic change of lung of Case 2. (**A**) Pulmonary alveoli are distented and filled with pink exudate. (**B**) Many lymphocytes and exfoliative cells fill the bronchioles. (**C**) Local enlargement of (**B**). There are no viral inclusion bodies in the epithelial cells of the bronchiole mucosa in the lung tissue. (**D**) Positive signal (red) of FHV-1 is primarily located in the cytoplasm of epithelial cells of bronchioles and exfoliative cells by immunohistochemistry stain. (**A**), (**B**), and (**C**) stained using haematoxylin and eosin (H&E). (**D**) stained by the mouse anti-FHV-1 antibody. Captured by Olympus CX 43 microscope and EPview Ver 1.2. www.olympus-sis.com.

During the necropsy, the bladder was found to be full and dilated (20 cm × 10 cm in diameter), holding 1050 mL of dark red urine. Congestion was presented on the surface of the bladder mucosa (S1 A and B). The renal pelvis of the right kidney showed effusion and dilation. The lungs appeared dull red, and there was swelling and liquid leakage present in the section analysed (Fig. 3). Moreover, there were no obvious abnormalities in other organs. A microscopic examination revealed coagulative necrosis of the massive glomeruli and tubules in the renal cortex, and bilirubin deposition in the tubule epithelial cells (S1C). The bladder had severe autolysis and the mucosa structure was disordered and homogeneous. The pulmonary alveoli were distended and filled with pink exudate. Many lymphocytes and exfoliative cells filled the bronchioles and no viral inclusion bodies were found in the epithelial cells of the bronchial mucosa. (Fig. 4A–C).

Owing to the previous fracture of the right leg, the activity of Case 2 was limited, which would have induced neurothlipsis of the urinary bladder^19^. This may have led to postrenal acute renal failure, which was supported by the symptoms of anuria. Therefore, we can conclude that the pathological diagnosis was postrenal acute renal failure owing to urine retention and interstitial pneumonia.

#### Case 3

Case 3 was a male snow leopard aged approximately 10 years. It was the last animal to exhibit sneezing and rhinorrhea, which occurred 5 days after symptom onset in Case 1. The animal recovered gradually after half a month of antiviral and supportive treatment, but died suddenly 5 months later. The body was not necropsied or otherwise examined.

### Detection of suspicious pathogens

According to the sequence of onset and the similarity of symptoms among the three snow leopards, infection with the causative agents of common respiratory infectious diseases were suspected, including CDV, FHV-1, FCV, mycoplasma, chalmydia. However, other lethal viral pathogens were also considered, including FIPV, FPLV, FeLV, SFTSV. Both FIPV and SFTSV can cause encephalitis and renal disease in cats^20–22^. The results of the detection of suspicious pathogens is shown in Table 1. All nasal swabs from the three snow leopards tested positive for FHV-1 and tested negative for the other suspicious pathogens, thus confirming FHV-1 as the only causative agent.

### FHV-1 distribution and location in tissues

To explore the relationship between FHV-1 infection and pathological changes, the distribution of FHV-1 in different tissues was determined. The FHV-1 was found in the lungs and nasal passages by PCR. The liver, spleen, kidney, and tonsils tissue samples were negative for the viruse (see Table 1). Immunohistochemistry results showed that the lungs of Case 2 were positive for FHV-1 and that the virus primarily located in the cytoplasm of epithelial cells of bronchioles and exfoliative cells (Fig. 4D).

### Viral isolation and detection

**Figure 5:**
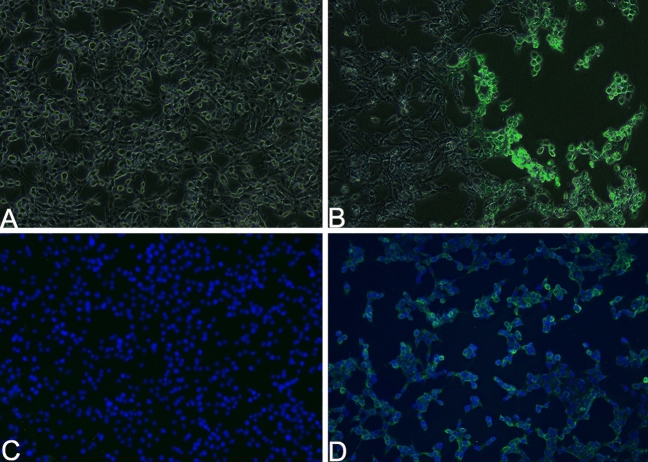
Isolation and identification of Feline herpesvirus type-1 (FHV-1) SL/QH/2019 strain. (**B**) FK81 cells inoculated with nasal swab supernatant of Case 2 for 36 h and show an obvious cytopathic effect (CPE), characterized by a round shape, pyknosis, fusion and aggregating like “fleece-pulling.” (**D**) The positive signal (green) of FHV-1 located in the cytoplasm of FK81 cells by indirect immunofluorescence. (**A**) and (**C**) Mock controls. (**A**) and (**B**) are merging image of bright-filed and fluorescence. (**C**) and (**D**) The nucleus is stained with DAPI and presents blue. FK81 cells from virus-free served as a negative control (×100). Captured by Nikon TS2-FL fluorescence microscope and NIS-Elements D Ver5.01.00. www.microscope.healthcare.nikon.com/products/software/nis-elements.

At 36 h post-inoculation, using the nasal swab supernatant of Case 2, FK81 cells showed an obvious cytopathic effect (CPE), characterized by a round shape, pyknosis, fusion, and aggregating like “fleece-pulling” (Fig. 5B) compared with the mock control (Fig. 5A). PCR revealed that cultures inoculated with the material from nasal swabs were positive for FHV-1. Using indirect immunofluorescence, most of the positive signal (green) of the presence of FHV-1 was shown to be located in the cytoplasm of FK81 cells (Fig. 5B,D) compared with the mock control (Fig. 5C). This finding showed that the FHV-1 isolate replicated in cells. These results further confirmed that the snow leopards were infected with FHV-1.

### Phylogenetic analysis based on gD and gE genes of FHV-1

**Figure 6:**
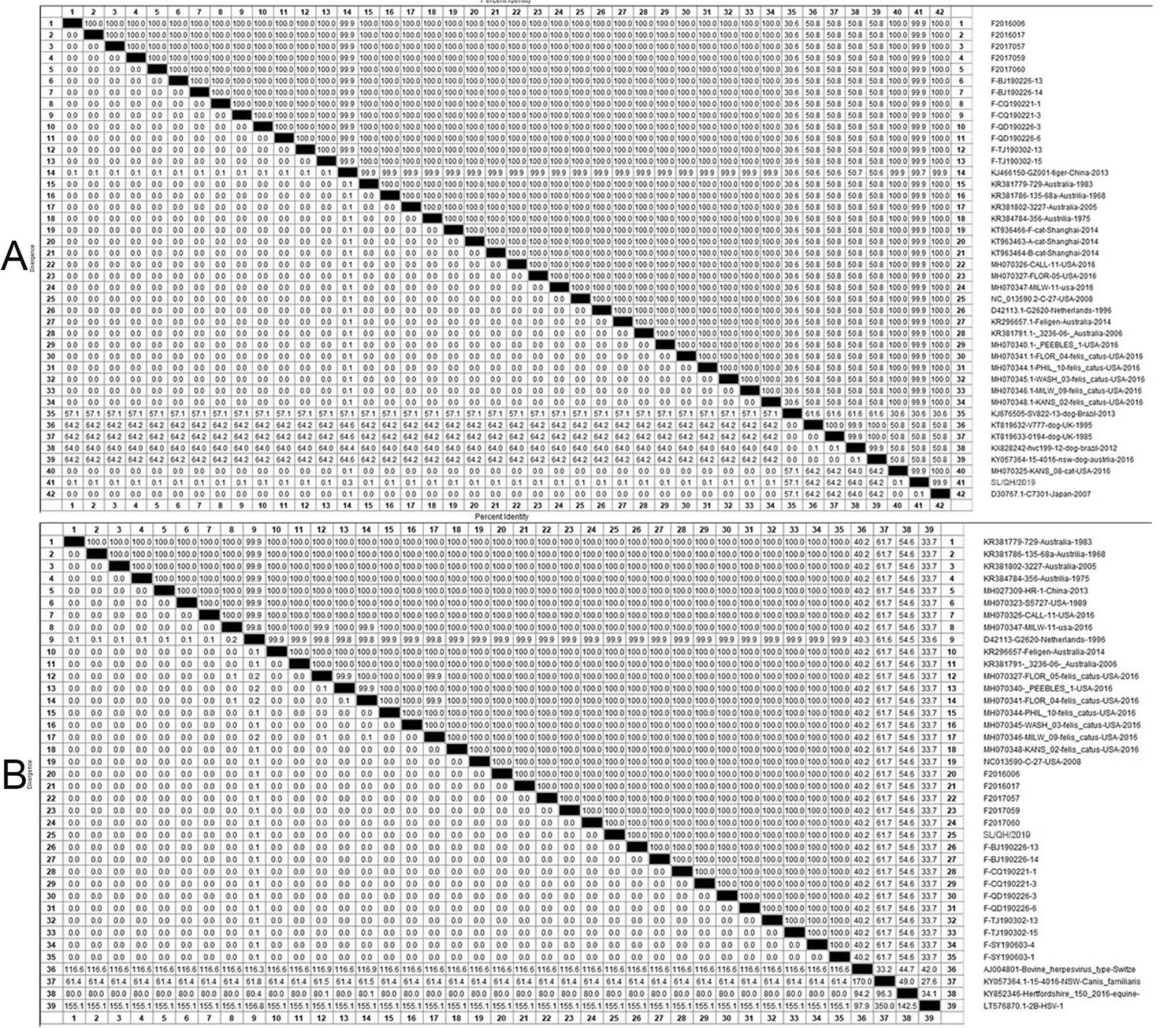
Alignment of the nucleotide sequences of FHV-1 gene. (**A**) Alignment of the gD gene of FHV-1. SL/QH/2019 share 99.9% identity with isolates of FHV-1 from cats and 99.7% identity with isolates of FHV-1 from tigers in China on the gD gene. While SL/QH/2019 share low identity (from 30.6 to 50.8%) with CHV-1 on the gD gene. (**B**) Alignment of the gE gene of FHV-1. SL/QH/2019 share 100% identity with isolates of FHV-1 from cats; however, low identity (from 27.6 to 61.7%) with CHV-1 on gE gene. ▲: The mainly epidemic isolates in China.

In this study, the three isolates shared 100% identity with the gD (GenBank accession number: OK087390) and gE genes; therefore, the FHV-1 isolates from snow leopards were named SL/QH/2019. According to the alignment, SL/QH/2019 shared 99.9% identity with isolates of FHV-1 from domestic cats and 99.7% identity with isolates of FHV-1 from tiger in China based on the gD gene. However, SL/QH/2019 shared a low degree of identity (from 30.6 to 50.8%) with canine herpesvirus type 1 (CHV-1) based on the gD gene (Fig. 6A).

Similarly, SL/QH/2019 shared 100% identity with isolates of FHV-1 from domestic cats; however, there was a low degree of low identity (from 27.6 to 61.7%) with CHV-1 based on the gE gene (Fig. 6B).

**Figure 7:**
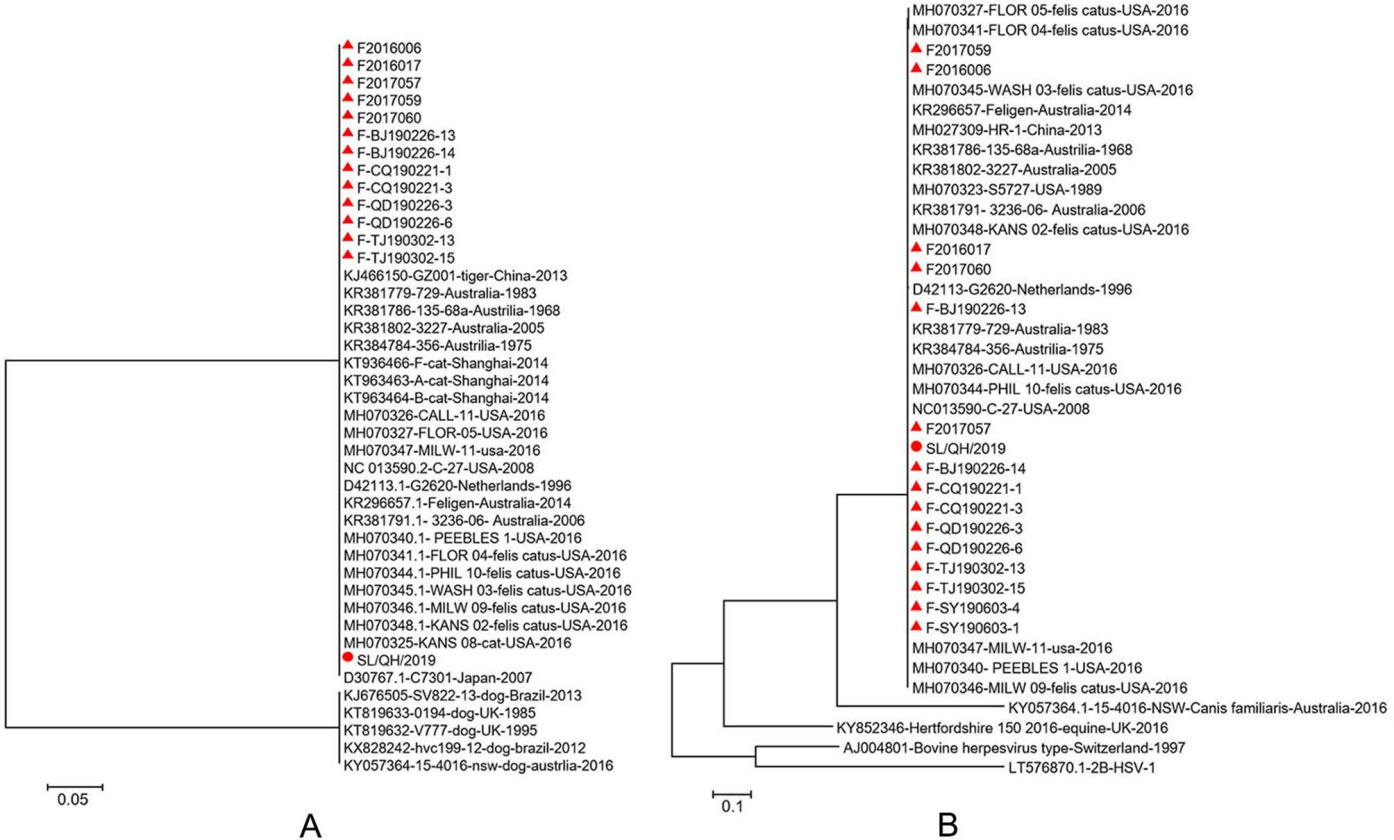
Phylogenetic tree based on the nucleotide sequences of the gD and gE gene of FHV-1. (**A**) Phylogenetic tree based on the nucleotide sequences of gD gene. (**B**) Phylogenetic tree based on the nucleotide sequences of gE gene. ▲: The mainly epidemic isolates in China. The trees are constructed with MEGA 7.0.

Specifically, SL/QH/2019 is highly homologous with primarily epidemic isolates (Fig. 7B▲) in China.

The phylogenetic tree based on the gD and gE gene sequences showed that the isolate investigated in this study was closely related to the isolates of FHV-1 from domestic cats (Fig. 7A,B), which is consistent with the alignment analysis.

## Discussion

FHV-1 in snow leopards has been found by next-generation sequencing using serum and rectal swab samples^5^; however, there has been no clinical case report about active infection and symptoms of FHV-1 infection in snow leopards. In this study, all of the snow leopards with obvious sialorrhea and sneezing symptoms were confirmed to be infected with FHV-1 using real-time qPCR. According to the clinical histories, FHV-1 can infect snow leopards and spread rapidly among them, causing clinical symptoms similar to those in domestic cats. However, acute death is rare in adult domestic cats^1^. The pathogenesis of FHV-1 infection is based on two different mechanisms^23^. The first mechanism is that FHV-1, as a cytolytic virus, can damage the epithelial cells of the mucosae and cornea, leading to ulceration. The second mechanism is an immune-mediated reaction driven by antigenic stimulation^23^.

In this study, FHV-1 infection first caused changes in the respiratory system and pneumonia of Case 2, based on the clinical symptoms and pathological examination. This result was consistent with those in previous reports in cats^24^. Unfortunately, because there were no fixed lung samples of Case 1 and Case 3, we could not compare the samples from these animals to those form Case 2. In Case 2, in addition to the characteristic morphological changes associated with interstitial pneumonia which was primarily caused by a viral infection^25^, immunohistochemistry and PCR also detected the FHV-1 in the lung tissue sample. FHV-1 is reported to target both respiratory epithelial cells and pneumocytes and kill the infectious cells via apoptosis or inducing neutrophil infiltration^3^. In our study, FHV-1 was mainly located in the epithelial cells of the bronchioles with limited neutrophil infiltration. However, a massive amount of necrotic cast-off cells were visible in the bronchioles. This fact also supports the assertion that the pathway of FHV-1 shedding is primarily the respiratory tract.

In addition to viral rhinotracheitis and pneumonia, FHV-1 infection also causes feline ocular disease and ulcerative dermatitis^3,7,26^. Therefore, the non-suppurative meningoencephalitis in Case 1 and renal damage in Case 2 were possibly caused by the infectious agent. Non-suppurative meningoencephalitis is frequently found in dogs and cats, and the causative agents may include FPLV, FIPV, FLeV, West Nile virus and SFTSV^20,21,27^. We did not detect FPLV, FIPV, FLeV and SFTSV; while West Nile virus has not been reported in China. It has been reported that one white-handed gibbon died of cerebral infarction and myocardial fibrosis with herpes simplex I and Epstein-Barr virus^28^. FHV-1 has also been reported as a causative agent of severe nonsuppurative meningoencephalitis in domestic cats^29^. Primary or virus-triggered secondary immune-mediated mechanisms cannot be ignored^27^. In this study, although FHV-1 was not detected using PCR in any organs other than the lungs of Case 1, the vascular cuff reactions and demyelinating lesions were generally suggestive of a viral aetiology affecting the brain^25^. Moreover, herpesvirus is a common causative agent in both humans and animals^30–32^. Thus, the FHV-1 infection may be related to the non-suppurative meningoencephalitis in Case 1.

To date, urinary system diseases associated with FHV-1 have not been reported. of the tests for kidney disease using PCR and immunohistochemistry to detect FHV-1 were negative. Our conclusion is that the kidney and urinary bladder pathology observed in this study were caused by an unrelated non-infectious factors.

For Case 3, since it was neither necropsied nor had any samples stored, the relationship between its death and FHV-1 infection is unclear. Unlikely Case 1 and Case 2, Case 3 had no underlying disease and recovered gradually from infection with treatment before death. However, the hallmark of reactivation of latency of alphaherpesvirus is worth considering^7^. When animals experience stress, viral reactivation readily occurs spontaneously suggesting that a link between its death and its previous infection cannot be ruled out^33^.

FHV-1 has only one serotype and is relatively genetically homogenous^1^. The gD protein probably has host selectivity and stimulates the host to produce high level of cellular immunity and anti-gD antibodies^34^. The gE protein is mainly related to the virulence of FHV-1^35^. The results showed that SL/QH/2019 was highly homologous with the main epidemic isolates from domestic cats in China. In the follow-up monitoring of stray cats around the zoo, some were found to carry FHV-1 (unpublished). Thus, the FHV-1 strain that infected the snow leopards probably originated from feral cats in the zoo. This shows that the genomic variation is not necessary for the cross-species transmission of FHV-1.

These cases show that the common FHV-1 from domestic cats is highly infective, pathogenic and fatal. Moreover some surveys show that the infection rate of FHV-1 is very high (40–50%) in feral cats^15^. Administrators of zoos and natural reserves should implement effective measures to prevent the FHV-1 carried by cats from reaching the wildlife. We suggest the following measures. (1) *Immunization*. Vaccination is a priority for the prevention of infectious diseases and should involve by vaccinating not only captive animals but also stray cats around zoos and reserves to build an “immune barrier”^36^. (2) *Regular monitoring* for symptoms to ensure infections are detected as soon as possible^37^. (3) *Removal* of stray cats from the park. (4) *Isolation*. Sick animals should be immediately removed and treated in an isolated area, with appropriate measures taken to limit the transfer of infection to other animals either directly or via the staff^38^. Due to the potential for the reactivation of latent FHV-1, recovered animals should be carefully monitored and considered for isolation during periods of stress. The above measures are applicable not only applicable to FHV-1 but also need to be considered for other infectious diseases from domestic animals. We recommend that zoos and natural reserves administrators take the risk posed by such diseases seriously and create action plans to limit the FHV-1 transmission risk from domestic cats.

## Materials and methods

### Case descriptions

Three dead snow leopards in the Qinghai-Tibet Plateau Wild Zoo that had presented with sneezing and rhinorrhea were numbered 1, 2, and 3. Their clinical information is shown in Table 1.

### Sample collection and pathological examination

After their deaths, necropsies were performed for Case 1 and 2. The details of samples are shown in S1.

The frozen samples were sent for PCR examination. The samples fixed with 4% neutral formalin were embedded in paraffin section and were stained using haematoxylin–eosin for pathological examination. Additionally, the fixed tissues were immunohistochemically stained with mouse anti-FHV-1 monoclonal antibody 4G12 (ProtTech, China), and observed under an optical microscope and photographed.

### PCR assays

We tested for the following pathogens: CDV, FHV-1, FPLV, FCV, mycoplasma, and chlamydia in nasal swabs, FIPV in spleen samples, and FeLV in faecal samples using T8 real-time fluorescence quantitative PCR instrument and the appropriate commercial kit (manufactured by Beijing Anheal Laboratories Co., Ltd. China). Vrial genomic RNA was extracted from the spleens and kidneys of Case 1 and 2 for the detection of SFTSV using PCR^16^.

Viral genomic DNA was extracted from nasal swabs and tissue samples using a DNA Viral Genome Extraction Kit (D2400, Solarbio, China) and subjected to PCR. The complete genome of the glycoprotein D (gD) gene and glycoprotein E (gE) gene of FHV-1 were amplified. The primer sequences and PCR conditions are shown in S2.

### Virus isolation

The nasal swab from Case 2 was processed, creating a supernatant using phosphate buffer solution (0.1 mol/L, pH 7.4, PBS) and filtered using a 0.22 μm filter membrane for sterilisation. The viral culture performed according to the method described by Zhang^39^. The F81 cells (Crandell Feline Kidney, purchased from Procell, China) were selected for the replication of FHV-1. F81 cells infected with FHV-1 were detected using PCR and indirect immunofluorescence using mouse anti-FHV-1 monoclonal antibody 5H8 (ProtTech, China).

### Sequence analysis and phylogenetic tree construction

The alignment of FHV-1 strains were analyzed based on the gD and gE genes using MegAlign (7.1). The phylogenetic trees were constructed based on the gD and gE genes using MEGA-7 software (7.0).

### Ethics statement

The authors confirm that the ethical policies of the journal, as noted on the journal’s author guidelines page, have been adhered to. All relevant guidelines for the use of animals in scientific studies were followed. The study did not include any experimentation on animals or humans, and samples were taken from natural dead animals that was approved by the owner of animal.

## Supplementary Information

Supplementary Information 1.

Supplementary Information 2.

Supplementary Information 3.
