# Supplementary material for: Feline herpesvirus infection and pathology in captive snow leopard
Source: Sci Rep. 2022 Apr 28;12:4989. doi: 10.1038/s41598-022-08994-4 (PMC9051049; doi:10.1038/s41598-022-08994-4)
Supplement: Supplementary file 1 — Supplementary Information 1. [file 41598_2022_8994_MOESM1_ESM.doc]

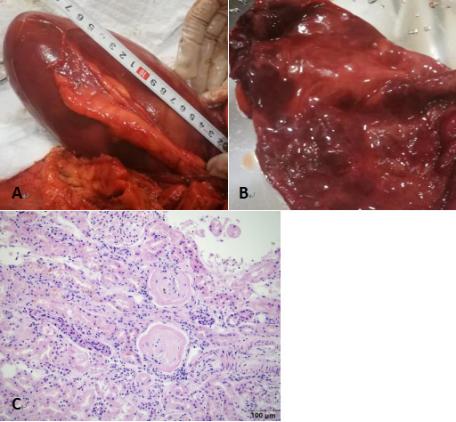


S1. Gross and microscopic observation of the urinary bladder and kidney of Case 2.

A. Urinary bladder. The bladder accumulates and dilates, 20 cm × 10 cm in diameter. B. Urinary bladder mucosa. The congestion presents on the surface of the urinary bladder mucosa. C. Kidney. Microscopic observation of kidney, coagulation necrosis of massive glomerulus and tubules in the renal cortex, and bilirubin deposition in tubule epithelial cells are visible (H&E). Captured by Olympus CX 43 microscope and EPview Ver 1.2. www.olympus-sis.com.
