# Supplementary material for: Feline herpesvirus infection and pathology in captive snow leopard
Source: Sci Rep. 2022 Apr 28;12:4989. doi: 10.1038/s41598-022-08994-4 (PMC9051049; doi:10.1038/s41598-022-08994-4)
Supplement: Supplementary file 2 — Supplementary Information 2. [file 41598_2022_8994_MOESM2_ESM.doc]

S1 The types and storage conditions of samples from three snow leopards

| Sample | Nasal swab | Tonsil | Lung | Heart | Liver | Spleen | Kidney | Brain |
| --- | --- | --- | --- | --- | --- | --- | --- | --- |
| Case 1 | s | s/f | s | s | s | s | s | s/f |
| Case 2 | s | n | s/f | s/f | s/f | s/f | s/f | n |
| Case 3 | s | n | n | n | n | n | n | n |

s: storage in -20℃; f: fixed by 4% neutral formalin; n: no collection
