# Supplementary material for: Feline herpesvirus infection and pathology in captive snow leopard
Source: Sci Rep. 2022 Apr 28;12:4989. doi: 10.1038/s41598-022-08994-4 (PMC9051049; doi:10.1038/s41598-022-08994-4)
Supplement: Supplementary file 3 — Supplementary Information 3. [file 41598_2022_8994_MOESM3_ESM.doc]

S2 Oligonucleotide primers, reaction systems and PCR conditions used for the amplification of gD and gE gene

| Target gene | Primer sequence | Length of fragment | Reaction systems | Conditions for PCR |
| --- | --- | --- | --- | --- |
| gD | Forward primer: 5’-AACTGCCCTCCATTCTACTC-3’  Reverse primer: 5’-TTGGTCCAGACTCCAACCTAT-3’ | 1269bp | 2× Taq PCR Mix, 12.5 μL  F/R primer, 1 μL  Template, 1 μL  ddH2O, 9.5 μL | Fore-denaturalization: 95℃, 3min.  Denaturalization: 94℃, 30s. Anneal:54℃, 30s. extension: 72℃, 1min20s. 30 circles, extension: 72℃ 10min. |
| gE | Forward primer: 5’-AGGTTTTATGCCCAGTTCACAGT-3’  Reverse primer: 5’-GAGTTTACGAGCAGCTTCCAATT-3’ | 1726bp | 2× Taq PCR Mix, 12.5 μL  F/R primer, 1 μL  Template, 1 μL  ddH2O, 9.5 μL | Fore-denaturalization: 95℃, 3min.  Denaturalization: 94℃, 30s. Anneal:55℃, 30s. extension: 72℃, 1min50s. 30circles, extension: 72℃ 10min. |
